# Relationship between depression, neuropsychiatric symptoms and cognitive profiles of the elderly in India: An assessment using the ICMR-MUDRA toolbox

**DOI:** 10.1017/gmh.2026.10246

**Published:** 2026-06-10

**Authors:** U. Venkatesh, Varkey Nadakkavukaran Santhosh, Margubur Rahaman, Om Prakash Bera, Ashoo Grover, Manoj Prithviraj, Hari Shanker Joshi, Anand Mohan Dixit, Vibha Dutta

**Affiliations:** 1 Department of Community Medicine & Family Medicine, All India Institute of Medical Sciences, Gorakhpur, Uttar Pradesh, India; 2 Global Health Advocacy Incubator, USA; 3 Delivery Research Division, Indian Council of Medical Research, New Delhi, India; 4 Department of Psychiatry, All India Institute of Medical Sciences, Gorakhpur, Uttar Pradesh, India; 5 Indian Council of Medical Research - National Institute of Health Research, Gorakhpur, Uttar Pradesh, India; 6 All India Institute of Medical Sciences, Gorakhpur, Uttar Pradesh, India

**Keywords:** cognition disorders, elderly, cognitive dysfunction, depression, neuropsychiatric symptoms

## Abstract

India’s aging population faces escalating cognitive decline and neuropsychiatric challenges. This cross-sectional study examined relationships between geriatric depression, neuropsychiatric symptoms and cognitive performance among 1,013 community-dwelling elderly participants (≥60 years) in Gorakhpur using the ICMR-MUDRA Toolbox. Multistage random sampling was employed. Depression was assessed using GDS-30, and neuropsychiatric symptoms were assessed using NPI-Brief. It was observed that geriatric depression was present in 63.3% of participants, while neuropsychiatric symptoms affected 78.5%, predominantly agitation (15%) and irritability (12%). Cognitive performance declined significantly across depression severity in attention, executive function, memory, language and visuospatial domains (*P* < 0.001). Adjusted regression revealed that mild depression was negatively associated with executive function and memory, while severe depression showed stronger associations with language and visuospatial abilities. Neuropsychiatric symptoms independently impacted cognitive domains. These findings highlight the need for integrated mental health screening and early intervention strategies within routine geriatric care in India.

## Impact Statements

India is experiencing rapid population aging, yet mental health conditions among older adults, particularly depression, behavioral symptoms and cognitive decline, often remain undetected and untreated. This study demonstrates that depression and neuropsychiatric symptoms are highly prevalent among community-dwelling older adults and are strongly linked to impairments across multiple cognitive domains, including memory, language, executive function and visuospatial abilities. By using the culturally and linguistically validated ICMR-MUDRA Toolbox in a real-world community setting, this research provides robust, India-specific evidence that mental health and cognitive decline in later life are deeply interconnected rather than isolated conditions. The findings have direct public health relevance. They support the integration of routine mental health and cognitive screening into Indian primary healthcare services, particularly at the community and primary health center levels, where most older adults seek care. Early identification of depression and neuropsychiatric symptoms may enable timely, low-cost interventions that help slow cognitive decline, preserve functional independence and improve quality of life for older adults. This research highlights the importance of culturally appropriate assessment tools in aging populations globally, especially in low- and middle-income settings. The study contributes evidence to inform geriatric care policies, training of frontline health workers and the design of integrated mental health programs aimed at promoting healthier and more dignified aging.

## Introduction

The global demographic transition has resulted in unprecedented population aging, with India experiencing one of the most rapid increases in elderly populations worldwide (Sathyanarayana Rao and Shaji, 2007). By 2050, India is projected to have over 300 million individuals aged 60 years and above, representing nearly 20% of the total population (Rajan et al., 2003). This demographic shift brings significant public health challenges, particularly concerning cognitive health and neuropsychiatric well-being among older adults. Cognitive decline and neuropsychiatric symptoms represent interconnected domains of mental health that substantially impact quality of life, functional independence and healthcare utilization in elderly populations (He et al., 2023). Depression, one of the most prevalent mental health conditions among older adults, affects ~10–15% of community-dwelling elderly individuals globally, with higher prevalence rates reported in developing countries, including India (Barua et al., 2011). Concurrently, neuropsychiatric symptoms such as agitation, apathy, irritability and sleep disturbances are increasingly recognized as significant contributors to cognitive impairment and functional decline in aging populations (You et al., 2015).

Depression in older adults is a common mental health condition characterized by persistent low mood, loss of interest and functional impairment, often accompanied by cognitive deficits such as reduced attention, memory and executive function (Yesavage et al., 1982; Rock et al., 2014). Cognitive profile refers to the pattern of performance across multiple cognitive domains, including attention, executive function, memory, language and visuospatial abilities, which collectively reflect an individual’s overall cognitive functioning and are critical for independent living (Menon et al., 2020; Verma et al., 2021). Neuropsychiatric symptoms encompass a range of behavioral and psychological disturbances, such as agitation, apathy, irritability, anxiety and sleep disturbances, which commonly co-occur with cognitive impairment and significantly impact quality of life and disease progression in older adults (Kaufer et al., 2000; Lyketsos et al., 2002).

The relationship between depression, neuropsychiatric symptoms and cognitive performance is complex and bidirectional. Depression in elderly individuals has been consistently associated with deficits across multiple cognitive domains, including executive function, attention, memory and processing speed (Rock et al., 2014). These cognitive changes, often termed “pseudodementia” or depression-related cognitive impairment, may precede or co-occur with neurodegenerative processes (Mars and Marwaha, 2026). Similarly, neuropsychiatric symptoms can both result from and contribute to cognitive decline, creating a cycle of deteriorating mental health and functional capacity (Krell-Roesch et al., 2021). Recent evidence from low- and middle-income countries highlights that late-life depression is strongly associated with accelerated cognitive decline and increased risk of dementia, underscoring the need for integrated mental health and cognitive screening in aging populations (Muhammad and Meher, 2021; Abdoli et al., 2022; Fan et al., 2024). A study from north India reported that 33.9% and 6.8% of elderly individuals experienced mild–moderate and severe depression, respectively, with female gender emerging as the only significant predictor; however, evidence such as this from Gorakhpur remains limited despite the rising burden of geriatric depression in rural north India (Sahni et al., 2020). Gorakhpur was selected as it represents a rural, resource-limited setting with limited access to geriatric mental health services.

In the Indian context, several factors compound these challenges. Cultural stigma surrounding mental health conditions often leads to underrecognition and inadequate treatment of depression and neuropsychiatric symptoms in elderly populations (Chakrapani and Bharat, 2023). Additionally, linguistic diversity, varying educational backgrounds and limited access to specialized healthcare services create barriers to accurate assessment and intervention. Traditional Western-developed cognitive assessment tools may not adequately capture the cognitive abilities of Indian elderly populations due to cultural and linguistic differences (Lakshminarayanan et al., 2022).

Recent research has highlighted the need for culturally appropriate and linguistically validated assessment instruments for evaluating cognitive function in diverse populations (Czerwinski-Alley et al., 2024). The Indian Council of Medical Research (ICMR) developed the MUDRA Multilingual Dementia Research and Assessment (MUDRA) Toolbox specifically to address these limitations (Verma et al., 2021; Venkatesh et al., 2026). Unlike conventional cognitive assessment tools, which are often developed in Western settings and may be influenced by language, education and cultural biases, the MUDRA Toolbox is specifically designed and validated for the Indian context. This comprehensive neuropsychological battery has been standardized across five major Indian languages and incorporates culturally relevant tasks, thereby improving diagnostic accuracy and reducing misclassification in heterogeneous populations (Menon et al., 2020). It enables context-sensitive assessment across multiple cognitive domains, including attention, executive function, memory, language and visuospatial abilities, making it particularly suitable for large-scale community-based studies in India.

Understanding the specific patterns of cognitive impairment associated with depression and neuropsychiatric symptoms is crucial for developing targeted interventions and improving diagnostic accuracy in elderly populations (Rock et al., 2014). The present study addresses these knowledge gaps by examining the relationship between neuropsychiatric symptoms, geriatric depression and cognitive performance across multiple domains in a large sample of community-dwelling elderly individuals in Gorakhpur, Uttar Pradesh. This gap reflects limited region-specific evidence from rural North India; Gorakhpur was selected as a representative resource-limited setting to capture regional variability and contextual determinants. The findings may contribute to improved understanding of cognitive aging patterns in Indian contexts.

The present study aligns with Sustainable Development Goal (SDG) 3 (Good Health and Well-being) by addressing the growing burden of late-life depression and its association with cognitive decline, which has been identified as a key contributor to disability and dementia risk in aging populations (Muhammad and Meher, 2021; Abdoli et al., 2022). Therefore, this study aimed to examine the relationship between neuropsychiatric and cognitive profiles among elderly individuals in Gorakhpur, Uttar Pradesh, using the validated ICMR-MUDRA Toolbox. The specific objectives of the study were to determine the prevalence of geriatric depression and neuropsychiatric symptoms, assess cognitive performance across key domains and examine the associations between depression severity, neuropsychiatric symptoms and cognitive function among community-dwelling older adults.

## Methods

### Study design and study setting

This research utilized a cross-sectional methodology and focused on elderly individuals residing in Gorakhpur, a city situated in Uttar Pradesh’s northeastern area within India. The data gathering phase occurred from March through September of 2023. The research findings were documented following the STROBE (Strengthening the Reporting of Observational Studies in Epidemiology) reporting standards. The study was carried out in Primary Health Centers (PHCs) predominantly located in rural areas of the Gorakhpur district, serving primarily rural populations.

### Ethical consideration and informed consent

The Institutional Human Research Ethics Committee of All India Institute of Medical Sciences, Gorakhpur, provided ethical approval (Ref no: HEC/AIIMS-GKP/BMR/107/2022) on 23 April 2022. Each participant received a comprehensive explanation of the research objectives. Before involvement, written consent was secured from all participants. The study exclusively included individuals who voluntarily consented to participate.

### Selection criteria

Participants meeting the inclusion criteria were individuals aged 60 years or older who held permanent residency status in the designated administrative divisions. Exclusion criteria applied to nonlocal inhabitants outside the elderly age range, as well as those with previous head injuries, infectious diseases and cerebrovascular accidents. Additionally, individuals with significant auditory or visual deficits that might interfere with evaluation procedures were omitted from the study.

### Sample size and sampling technique

Sample size calculation was performed utilizing the OpenEpi calculator (Version 3.0), drawing from previous research conducted by Pandit et al. (2024) that documented cognitive impairment prevalence at 24.9% within India’s rural communities. With an alpha error of 5% and statistical power set at 80%, the computed minimum sample requirement was 288 participants. Due to the multistage random sampling methodology employed, a design effect factor of three was incorporated, resulting in an adjusted sample of 864 individuals. A design effect of three was applied to account for clustering at the PHC level and to ensure adequate statistical power under the multistage sampling design. The final target was established at 1,000 participants to ensure sufficient representation and compensate for potential nonparticipation rates.

**Figure 1.**
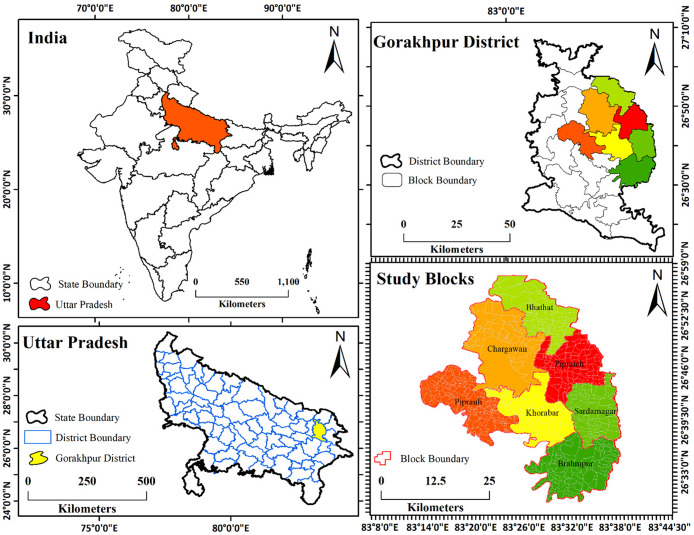
Geographical representation of the study area. The top-left panel shows the location of Uttar Pradesh (highlighted in red) within India. The bottom-left panel highlights Gorakhpur district (in yellow) within Uttar Pradesh. The top-right panel illustrates the block boundaries within Gorakhpur district. The bottom-right panel presents the study blocks within Gorakhpur district, with different colors representing distinct blocks. Figure 1. long description.

A multistage random sampling technique was utilized for selecting both study sites and participants. During the initial phase, seven administrative blocks were randomly chosen from the total 20 available, functioning as primary sampling units. Following this, one Primary PHC was randomly selected from each chosen block. In the concluding phase, qualified participants were systematically enrolled from the designated PHCs using a random number table method to ensure unbiased selection at each stage. Figure 1 displays a geographical representation of the research area.

### Instrument design and pilot testing

This research employed the ICMR-MUDRA Toolbox, a validated instrument designed for linguistic and cultural suitability across five primary Indian languages (Menon et al., 2020; Verma et al., 2021). The Hindi adaptation of this tool was implemented to evaluate various cognitive areas, encompassing overall cognition, attention and executive functions, episodic memory, language capabilities and visuospatial skills.

Assessment of attention and executive functions involved the Trail Making Test (TMT), Category Fluency Test and Phonemic Fluency Test. Episodic memory was evaluated using the Verbal Learning Test (VLT) and the Test des Neuf Images (TNI-93). Language abilities were assessed through the Picture Naming Test in combination with the Frenchay Aphasia Screening Test (FAST). Visuospatial skills were measured using the Modified Taylor Complex Figure Test (MTCF) and the Line Bisection Test. The TMT utilized time-based measurements (in seconds), while all other assessments were scored using standardized point-based systems (Venkatesh, Santhosh, Grover, et al. 202 6).

Geriatric depression and neuropsychiatric symptoms are considered key explanatory variables. Geriatric depression was assessed using the 30-item Geriatric Depression Scale (GDS-30), a validated yes/no questionnaire designed for older adults. Administered verbally, one point is scored for each depressive response. Scores of 10–19 indicate mild depression, and 20–30 indicate severe depression, as per standard scoring guidelines (Yesavage et al., 1982). Neuropsychiatric symptoms were recorded as a binary variable (presence vs. absence) using the caregiver-based questionnaire Neuropsychiatric Inventory–Brief (NPI-Brief). A composite binary variable indicating the presence of neuropsychiatric problems was created based on the summation of nine individual symptoms: delusions, hallucinations, euphoria, apathy, disinhibition, irritability, aberrant motor behavior, nighttime behavioral disturbances and appetite abnormalities (Kaufer et al., 2000). Each symptom was coded as present or absent. The total symptom count (range: 0–9) was computed and recoded into a binary variable, where 0 indicated no neuropsychiatric symptoms, and 1 indicated the presence of one or more symptoms.

To ensure consistency in the administration of the MUDRA Toolbox, five field investigators underwent comprehensive training and calibration under the guidance of subject matter experts, including standardization exercises and pilot assessments before data collection. Inter-rater reliability assessment of the MUDRA Toolbox employed Cohen’s kappa statistic, achieving a coefficient value of 0.88. A pilot study encompassing 50 participants was conducted within an administrative block excluded from the main research locations in Gorakhpur. The goal was to evaluate participant engagement with the MUDRA Toolbox and determine the feasibility of the study protocol. Information gathered during the preliminary investigation was omitted from the final analysis.

### Psychometric properties of the instruments

The tools used in this study have established reliability and validity in geriatric populations. The GDS-30 demonstrates high internal consistency (Cronbach’s *α*: 0.80–0.94) with good sensitivity and specificity for detecting depression in older adults (Yesavage et al., 1982). The NPI-Brief shows strong inter-rater reliability and good convergent validity with the full-scale instrument, supporting its use in community-based assessments (Kaufer et al., 2000). The ICMR-MUDRA Toolbox has been validated across diverse Indian populations, demonstrating good construct validity and reliability in assessing multiple cognitive domains while minimizing cultural and linguistic bias (Menon et al., 2020; Verma et al., 2021).

### Data collection

**Figure 2.**
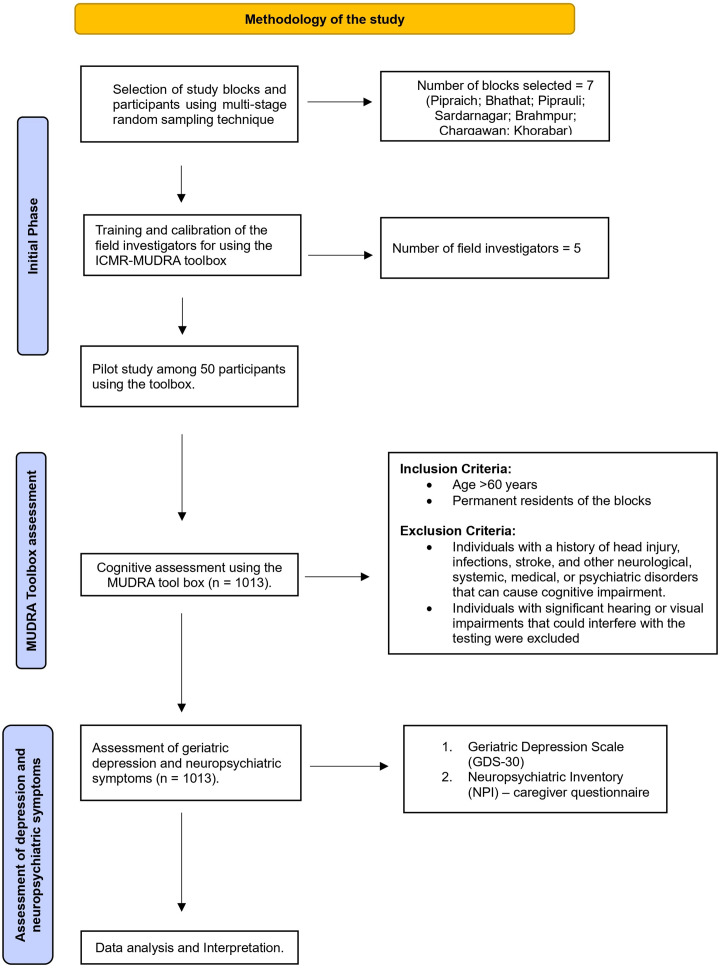
Flow diagram illustrating the methodology of the study. Figure 2. long description.

Every participant underwent a 90-min face-to-face cognitive assessment at the designated PHCs, evaluating multiple components of the MUDRA Toolbox. Standardized protocols were implemented across all research sites, with periodic calibration training conducted for examiners to ensure precision and consistency. Scoring procedures adhered to predetermined standards customized for each distinct MUDRA Toolbox domain. Subsequently, participants were evaluated using the GDS-30 and the caregiver-administered NPI-Brief questionnaire to measure geriatric depression and neuropsychiatric symptoms. In all cases, the NPI-Brief questionnaire was completed by a family member accompanying the participant during the PHC visit. All cognitive assessments were administered in a standardized sequence in accordance with the protocol of the ICMR-MUDRA Toolbox to ensure consistency and minimize potential order effects on participant performance. Quality control was ensured through periodic monitoring of field data collection, along with regular supervision and validation checks (Figure 2).

### Data processing and analysis

A composite TMT score was created by combining completion time and number of errors to reflect overall cognitive performance. Both measures were standardized using z-scores to account for differences in scale and then summed. Higher composite scores indicate poorer cognitive performance, reflecting slower task completion and more errors. Descriptive statistics were used to present the background characteristics of the participants. In particular, proportions with 95% confidence intervals (CIs) were calculated. The normality of cognitive variables was assessed using the Shapiro–Wilk test. All cognitive measures demonstrated significant deviation from normal distribution (*P* < 0.001) (Supplementary Table 1). Therefore, nonparametric statistical tests (Kruskal–Wallis *H* test and Mann–Whitney *U* test) were employed to assess the differences in cognitive performance by geriatric depression and neuropsychiatric symptom status. The Kruskal–Wallis *H* statistic is calculated using the following equation:

$$H=\frac{12}{N\;\left(N+1\right)}\sum_{j=1}^{k}\mathrm{nj}\;{\left(\bar{R}j-\bar{R}\right)}^{2}$$

The Mann–Whitney *U* test is calculated using the following equation:

$$U=n_{1}n_{2}+\frac{n_{1}\;\left(n_{1}+1\right)}{2}-R_{1}$$

$$z=\frac{U-\mu_{U}}{\sigma_{U}},\;\text{where},\;\mu_{U}=n_{1}n_{2},\sigma_{U}=\sqrt{\frac{n_{1}n_{2}\;\left(n_{1}+n_{2}+1\right)}{12}}$$

Adjusted and adjusted likelihood of cognitive outcomes by geriatric depression and neuropsychiatric symptoms were estimated using a generalized linear regression model.

$$E\left(Y_{i}\right)=\beta_{0}+\beta_{1}{\text{Mild}}_{i}+\beta_{2}{\text{Severe}}_{i}+\beta_{3}{\mathrm{NSP}}_{i}\;\sum_{k=3}^{p}\beta_{k}X_{\mathrm{ki}}+\varepsilon_{i}$$

where *Y_i_* is the cognitive outcome for individual *i*, Mild*_i_* and Severe*_i_* are dummy variables for depression status (reference: Normal), *X_ki_* denotes covariates (e.g., neuropsychiatric symptoms, age, sex and education) and *ϵ_i_* is the residual error term. Beta coefficients are reported with 95% confidence intervals. All tests were two-sided, and *p*-values <0.05 were considered statistically significant. Cognitive outcomes were adjusted for key covariates, including age, gender, education, occupation, per capita income (tertiles), diet type, tobacco use, alcohol consumption, hypertension and respiratory disease, to account for potential confounding effects of sociodemographic factors on neuropsychological performance (Muhammad and Meher, 2021; Pandit et al., 2024). These covariates were selected based on prior literature and their known influence on cognitive performance and mental health outcomes in older adults, thereby minimizing potential confounding in the analysis. Descriptive and regression analyses were conducted using STATA 17.0.1. Graphical outcomes constructed using R version 4.5.0.

## Results

### Participant characteristics

A total of 1,013 elderly participants were included in the study. The majority were male (58.7%, *n* = 595) compared to females (41.3%, *n* = 418). Most participants were aged 60–64 years (58.6%, *n* = 594), followed by 65–69 years (29.2%, *n* = 296). Regarding education, 84.1% (*n* = 852) had a primary school education. The predominant occupations were house makers (30.5%, *n* = 309) and clerical/shop/farmer (30.2%, *n* = 306) (Table 1).

**Table 1.** Demographic profile of the participants (*n* = 1,013) Table 1. long description.

Demographic profile	*n* (%)
***Gender***	
Male	595 (58.7%)
Female	418 (41.3%)
***Age***	
60–64 years	594 (58.6%)
65–69 years	296 (29.2%)
70–74 years	76 (7.5%)
>75 years	47 (4.6%)
***Education***	
Illiterate	17 (1.7%)
Primary school	852 (84.1%)
Middle school	37 (3.7%)
High school	65 (6.4%)
Graduate or above	42 (4.1%)
***Occupation***	
Clerical/shop/farmer	306 (30.2%)
Daily wager	237 (23.4%)
House maker	309 (30.5%)
Retired	54 (5.3%)
Unemployed	107 (10.6%)
**Per capita *income (INR)***	
≤3,250	338 (33.4)
3,250–5,000	446 (44.0)
>5,000	229 (22.6)
***Diet type***	
Vegetarian	358 (35.3)
Nonvegetarian	655 (64.7)
***Tobacco use***	
Yes	380 (37.5)
No	633 (62.5)
***Alcohol consumption***	
Yes	80 (7.9)
No	933 (92.1)
***Hypertension***	
Yes	112 (11.1)
No	901 (88.9)
***Respiratory disease***	
Yes	52 (5.1)
No	961 (94.9)

*Note:* All values are expressed as frequency with percentages (in parentheses).

### Prevalence of neuropsychiatric symptoms and depression

Based on the Geriatric Depression Scale, 36.7% of participants were classified as normal, 37.6% had mild depression and 25.7% had severe depression. Neuropsychiatric symptoms were present in 78.5% of participants, while 21.5% showed no symptoms (Supplementary Figure 1).

Individual neuropsychiatric symptom prevalence varied considerably (Supplementary Figure 2). The most common symptoms were agitation (15%) and irritability (12%). Anxiety was present in 7% of participants, while disinhibition affected 6%. Less prevalent symptoms included delusions and apathy (5% each), nighttime behavior disorders (4%), euphoria and appetite/eating disorders (3% each) and hallucinations and aberrant motor behavior (2% each).

### Cognitive performance across depression severity

Significant differences in cognitive performance were observed across geriatric depression categories (Table 2). Overall, increasing depression severity was associated with poorer performance across multiple domains. In attention and executive function, phonemic and category fluency scores declined significantly with higher depression levels (*P* < 0.05). Episodic memory showed consistent reductions, with significant declines in free recall, delayed recall and recognition tasks (*P* < 0.001). Language abilities were notably impaired, particularly in the Frenchay Aphasia Screening Test (*P* < 0.001), while visuospatial performance also declined significantly, as reflected in Line Bisection Test scores (*P* < 0.001).

**Table 2.** Comparison of cognitive Performance across Geriatric depression status Table 2. long description.

Geriatric depression status	Mean ± SD	Median [IQR]	Rank sum	H	p
***(A) Attention and executive function***					
***Trail making test – Part A errors***					
*Normal*	3.12 ± 4.56	2 [1–4]	181,552	3.73	0.155
*Mild depression*	3.45 ± 5.02	2 [1–5]	193,485		
*Severe depression*	3.87 ± 5.21	3 [1–6]	138,555		
***Trail making test – Part B errors***					
*Normal*	6.73 ± 6.39	2 [1–4]	203,399	11.23	0.004*
*Mild depression*	5.70 ± 5.50	2 [1–5]	182,885		
*Severe depression*	6.08 ± 6.00	3 [1–6]	127,308		
***Category fluency test***					
*Normal*	12.62 ± 5.71	12 [9–16]	199,535	8.3	0.016*
*Mild depression*	12.40 ± 5.25	12 [9–15]	192,208		
*Severe depression*	11.52 ± 6.12	11 [8–14]	121,849		
***Phonemic fluency test***					
*Normal*	9.78 ± 3.43	10 [8–12]	213,776	31.45	<0.001*
*Mild depression*	8.69 ± 3.91	8 [6–11]	177,462		
*Severe depression*	8.79 ± 4.34	8 [6–11]	122,354		
***(B) Episodic memory***					
***Verbal learning test (Free recall)***					
*Normal*	17.37 ± 3.46	18 [15–20]	227,604	76.91	<0.001*
*Mild depression*	15.18 ± 3.72	15 [12–18]	166,589		
*Severe depression*	15.00 ± 4.80	15 [11–18]	119,399		
***Verbal learning test (Delayed recall)***					
*Normal*	4.73 ± 1.44	5 [4–6]	215,164	40.76	<0.001*
*Mild depression*	4.13 ± 1.53	4 [3–5]	170,030		
*Severe depression*	4.49 ± 1.99	5 [3–6]	128,398		
***Verbal learning test (Delayed recognition)***					
*Normal*	13.87 ± 5.64	15 [10–18]	225,119	68.62	<0.001*
*Mild depression*	11.21 ± 5.62	11 [7–15]	174,844		
*Severe depression*	10.86 ± 5.74	10 [6–15]	113,629		
***TNI–93 test –* Total recall**					
*Normal*	13.28 ± 4.07	13 [10.47–15.95]	224,978	76.829	<0.001*
*Mild depression*	11.33 ± 3.98	11 [8.17–13.54]	181,342		
*Severe depression*	10.43 ± 4.01	10 [8.02–12.55]	107,270		
***TNI–93 test – Spatial* recall**					
*Normal*	6.33 ± 2.18	6 [5–8]	213,965	36.47	<0.001*
*Mild depression*	5.40 ± 2.10	5 [4–7]	171,110		
*Severe depression*	5.77 ± 2.17	6 [4–7]	128,517		
***(C) Language test***					
***Picture naming test***					
*Normal*	28.31 ± 3.17	29 [27–30]	208,343	24.91	<0.001*
*Mild depression*	27.29 ± 4.12	28 [25–30]	187,158		
*Severe depression*	26.35 ± 4.96	27 [23–30]	118,090		
***Frenchay aphasia screening test***					
*Normal*	17.58 ± 4.00	18 [15–21]	245,353	200.19	<0.001*
*Mild depression*	14.80 ± 4.00	15 [12–18]	182,192		
*Severe depression*	12.26 ± 4.57	12 [9–15]	86,047		
***(D) Visuo-spatial function***					
***Modified Taylor complex figure test***					
*Normal*	11.10 ± 12.44	5.86 [5.32–11.18]	197,394	11.653	0.029*
*Mild depression*	10.41 ± 12.13	6.28 [4.77–11.05]	196,073		
*Severe depression*	6.99 ± 8.80	4.64 [2.61–7.25]	119,110		
***Line bisection test***					
*Normal*	15.44 ± 3.68	16 [13–18]	220,452	66.111	<0.001*
*Mild depression*	14.19 ± 4.48	15 [11–17]	181,799		
*Severe depression*	13.59 ± 4.59	14 [10–16]	111,341		

*Note:* Values are expressed in mean ± standard deviation and median [inter-quartile range]. Statistical test used: Kruskal**–**Wallis test; *p ≤ 0.05 is considered statistically significant.

### Cognitive performance and neuropsychiatric symptoms

Comparison of cognitive performance based on neuropsychiatric symptom presence revealed selective impairments (Table 3). The category Fluency Test showed significant differences (*P* = 0.001), with participants in the group without symptoms scoring higher (12.72 ± 5.25) than those with symptoms (10.73 ± 6.67). Verbal Learning Test free recall was significantly better in the participants with symptoms (16.57 ± 3.63) compared to those without (15.75 ± 4.18, *P* = 0.006). Modified Taylor Complex Figure test demonstrated significant impairment in the presence of neuropsychiatric symptoms (7.57 ± 10.72) than in their absence (10.43 ± 11.77, *P* = 0.002).

**Table 3.** Comparison of cognitive performance across neuropsychiatric symptoms Table 3. long description.

Neuropsychiatric status	Mean ± SD	Median [IQR]	Rank sum	Z	p
***(A) Attention and executive function***					
***Trail Making Test – Part A errors***					
*Absent*	2.67 ± 3.97	2 [0–4]	400,134	−0.195	0.846
*Present*	2.52 ± 3.45	2 [0–4]	110,422		
***Trail making test – Part B errors***					
*Absent*	6.26 ± 6.26	4 [2–8]	393,748	−1.885	0.059
*Present*	5.91 ± 4.85	4 [2–8]	116,808		
***Category fluency test***					
*Absent*	12.72 ± 5.25	12 [9–16]	413,655	3.372	0.001*
*Present*	10.73 ± 6.67	10 [6–14]	96,901		
***Phonemic fluency test***					
*Absent*	9.19 ± 4.02	9 [6–12]	404,122.50	0.861	0.389
*Present*	8.85 ± 3.39	9 [6–11]	106,432.50		
***(B) Episodic memory***					
***Verbal learning test (Free recall)***					
*Absent*	15.75 ± 4.18	16 [13–19]	390,410.50	−2.755	0.006*
*Present*	16.57 ± 3.63	17 [14–19]	120,144.50		
***Verbal learning test (Delayed recall)***					
*Absent*	4.41 ± 1.66	5 [3–6]	395,842.50	−1.346	0.178
*Present*	4.56 ± 1.62	5 [3–6]	114,712.50		
***Verbal learning test (Delayed recognition)***					
*Absent*	11.67 ± 5.67	12 [7–16]	387,090	−3.659	0.058
*Present*	13.55 ± 6.08	14 [9–18]	123,465		
***TNI–93 Test –* Total recall**					
*Absent*	11.47 ± 4.08	11 [9–14]	382,897.50	−4.844	0.060
*Present*	12.98 ± 4.32	13 [10–16]	127,657.50		
***TNI–93 Test – Spatial* recall**					
*Absent*	5.70 ± 2.09	6 [4–7]	385,945	−3.962	0.078
*Present*	6.30 ± 2.42	6 [5–8]	124,610		
***(C) Language test***					
***Picture naming test***					
*Absent*	27.25 ± 4.25	26 [24–30]	392,574	−2.324	0.066
*Present*	28.00 ± 3.58	27 [25–30]	117,981		
***Frenchay aphasia screening test***					
*Absent*	14.99 ± 4.76	27 [25–30]	391,759	−2.398	0.096
*Present*	15.80 ± 4.19	13 [11–18]	118,796		
***(D) Visuo-spatial function***					
***Modified Taylor complex figure test***					
*Absent*	10.43 ± 11.77	10 [1–11]	411,091	3.148	0.002*
*Present*	7.57 ± 10.72	6 [0–6]	98,454		
***Line bisection test***					
*Absent*	14.44 ± 4.38	13 [12–17]	396,958	−1.127	0.260
*Present*	14.65 ± 4.00	14 [12–18]	113,597		

*Note*: Values are expressed in mean ± standard deviation and median [inter-quartile range]. Statistical test used: Mann**–**Whitney U test; *p ≤ 0.05 is considered statistically significant.

### Regression analysis

Adjusted regression coefficients revealed differential impacts of depression severity and neuropsychiatric symptoms on cognitive domains (Table 4). For geriatric depression categories (Model II), mild depression showed significant negative associations with multiple cognitive measures: TMT B (*β* = −0.20), phonemic fluency (*β* = −0.07) and several memory tasks, including VLT free recall (*β* = −0.12), delayed recall (*β* = −0.12) and delayed recognition (*β* = −0.21), along with additional significant impairments in TNI-93 total recall (*β* = −0.13), spatial recall (*β* = −0.14), FAST (*β* = −0.14) and line bisection test (*β* = −0.06). Severe depression demonstrated stronger negative associations, particularly with language functions: FAST (*β* = −0.30) and visuo-spatial function: MTCF (*β* = −0.33), along with significant impairments in TMT B (*β* = −0.16), phonemic fluency (*β* = −0.08), VLT free recall (*β* = −0.10), delayed recognition (*β* = −0.22), TNI-93 total recall (*β* = −0.20), spatial recall (*β* = −0.06), PNT (*β* = −0.06) and line bisection test (*β* = −0.09). Neuropsychiatric symptoms (Model II) showed significant negative associations with category fluency (*β* = −0.13), delayed recognition (*β* = −0.14), FAST (*β* = −0.05) and Modified Taylor Complex Figure test (*β* = −0.29) after adjusting for demographic and clinical variables.

**Table 4.** Unadjusted and adjusted coefficient of cognitive performance by geriatric depression scale category and neuropsychiatric symptoms Table 4. long description.

	(A) Attention and executive function				(B) Episodic memory					(C) Language Test		(D) Visuo-spatial function	
	TMT A	TMT B	Category fluency	Phonemic fluency	VLT (Free recall)	VLT (Delayed recall)	VLT (Delayed recognition)	TNI-93 *–* total recall	TNI-93 *–* spatial recall	PNT	FAST	MTCF	Line bisection test
**Geriatric depression scale category (Model I)**													
Mild depression	0.08	−0.17*	−0.02	−0.12*	−0.14*	−0.13*	−0.21*	−0.16*	−0.16*	−0.04*	−0.17*	−0.06	−0.08*
Severe depression	0.17	−0.10	−0.09*	−0.11*	−0.15*	−0.05	−0.25*	−0.24*	−0.09*	−0.07*	−0.36*	−0.46*	−0.13*
**Geriatric depression scale category (Model II)**													
Mild depression	0.03	−0.20*	0.02	−0.07*	−0.12*	−0.12*	−0.21*	−0.13*	−0.14*	−0.04*	−0.14*	−0.02	−0.06*
Severe depression	0.04	−0.16*	−0.07	−0.08*	−0.10*	−0.01	−0.22*	−0.20*	−0.06*	−0.06*	−0.30*	−0.33*	−0.09*
**Neuropsychiatric symptoms (Model I)**													
Present	−0.06	−0.06	−0.17*	−0.03	0.07	0.05	0.16	0.07	0.10	0.04*	−0.08	−0.32*	0.05
**Neuropsychiatric symptoms (Model II)**													
Present	−0.12	−0.09	−0.13*	−0.02	0.05	0.04	−0.14*	0.10	0.02	0.03	−0.05*	−0.29*	0.02

*Note*: TMT, Trail Making Test; VLT, Verbal Learning Test; PNT, Picture Naming Test; FAST, Frenchay Aphasia Screening Test; MTCF, Modified Taylor Complex Figure test. Statistical test used: Generalized linear regression model. All values are expressed beta coefficients. Model I Unadjusted; Model II are adjusted for age group, gender, education, occupation, per capita household monthly income (tertiles), diet type, tobacco consumption, alcohol consumption, hypertension, and respiratory disease. Coefficients represent log-transformed relative changes in cognitive test scores compared with the reference category, *p ≤ 0.05 is considered statistically significant.

Cognitive performance declined significantly with increasing depression severity across multiple domains, while neuropsychiatric symptoms were associated with selective impairments, particularly in executive function and visuospatial abilities; adjusted analyses confirmed these independent negative associations.

### Sociodemographic variations in cognitive performance

Adjusted analyses revealed important sociodemographic variations in cognitive performance across domains. Age-related patterns were evident, with participants aged ≥70 years demonstrating significant declines in phonemic fluency (*β* = −0.12), TNI total recall (*β* = −0.09) and line bisection scores (*β* = −0.06), while executive function measures did not show consistent age gradients. Gender differences were limited but domain-specific, with female participants showing lower TNI total recall scores (*β* = −0.11) compared to males, whereas other domains did not exhibit consistent variation. Educational attainment showed a strong and consistent positive association with cognitive performance across nearly all domains; individuals with higher education demonstrated significantly better outcomes in memory, fluency, naming and language measures, including delayed recognition (*β* = 0.66), TNI total recall (*β* = 0.30) and FAST scores (*β* = 0.73). Other covariates demonstrated selective associations, with higher income associated with slightly lower immediate recall (*β* = −0.05), nonvegetarian diet linked to lower memory and TNI scores and the absence of tobacco use associated with better recognition performance (*β* = 0.11).

## Discussion

The present study examined the relationships between geriatric depression, neuropsychiatric symptoms and cognitive profiles among community-dwelling elderly individuals in Gorakhpur, Uttar Pradesh, using the culturally adapted ICMR-MUDRA Toolbox. The findings reveal significant associations between geriatric depression, neuropsychiatric symptoms and multi-domain cognitive impairment, contributing important insights to the growing body of literature on cognitive aging in Indian contexts.

The study demonstrated a substantial burden of geriatric depression, with a considerable proportion of participants exhibiting both mild and severe depressive symptoms. Neuropsychiatric symptoms were even more widespread, with agitation and irritability emerging as the most common manifestations. Greater depression severity and the presence of neuropsychiatric symptoms were associated with marked declines across multiple cognitive domains, including attention, executive function, episodic memory, language and visuospatial abilities. Regression analyses further reinforced these findings, showing persistent negative associations, particularly in phonemic fluency, memory, language and visuospatial construction, even after adjusting for demographic variables.

The prevalence of geriatric depression observed in this study aligns with but exceeds several estimates from regional Indian investigations, highlighting potential regional variations influenced by socioeconomic and cultural factors. For instance, a meta-analysis encompassing 56 community-based studies across India reported a pooled prevalence of 34.4% among elderly individuals aged 60 years and above, with higher rates in rural settings at 41.7%. This analysis noted significant heterogeneity due to differences in screening tools and study designs, yet the current findings from a rural–urban mix in Uttar Pradesh suggest a heavier burden, possibly attributable to limited mental health resources and stigma in northern India (Pilania et al., 2019). Another systematic review and meta-analysis of 20 studies estimated a combined prevalence of 47%, emphasizing female preponderance and the impact of comorbidities, which resonates with the gender distribution in the present study (Bincy, et al. 2024). In a specific North Indian context, a cross-sectional study in rural areas found 40.7% prevalence using the Geriatric Depression Scale, similar to the instrument employed here, but still lower than the 63.3% reported (Sahni et al., 2020).

Current evidence suggests that cognitive impairment, depression and neuropsychiatric symptoms among older adults demonstrate significant regional variability influenced by sociocultural, economic and healthcare access factors in India. Studies from India and other low- and middle-income countries have highlighted substantial heterogeneity in prevalence and symptom profiles across regions, shaped by determinants such as education, rural–urban disparities, social support systems, comorbidities and access to healthcare services (Pilania et al., 2019; Muhammad and Meher, 2021; Sundarakumar et al., 2023). For instance, large-scale epidemiological analyses have shown that regional and contextual factors, including socioeconomic deprivation and cultural perceptions of mental health, significantly influence both the expression and reporting of depressive and neuropsychiatric symptoms, thereby affecting cognitive outcomes (Chakrapani and Bharat, 2023).

Globally, the prevalence of depression among older adults is generally lower than in India, reflecting differences in healthcare access, social support systems and economic stability. A systematic review and meta-analysis of studies worldwide estimated a global prevalence of 35.1% (95% CI: 30.2–40.4%), with variations by continent and methodology, but highlighting a consistent rise with age (Cai et al., 2023). Another meta-analysis reported 13.3% for major depressive disorder in older adults, with higher rates in women (11.9%) than men (9.7%), aligning with the gender patterns observed here but indicating a lower overall burden in high-income countries (Abdoli et al., 2022). The World Health Organization estimates that 14% of adults aged 60 years and over live with mental disorders, including depression, contributing to 10.6% of total years lived with disability in this group (Mental Health of Older Adults, 2025). These global figures contrast with the elevated rates in the present Indian sample, possibly due to factors like poverty, familial disruptions from migration and inadequate geriatric care in low- and middle-income countries, as noted in comparative analyses.

Among the Indian studies, the prevalence of neuropsychiatric symptoms among the elderly population in the present study is high. A study by Sundarakumar et al., assessing neuropsychiatric conditions in parallel aging cohorts in India, reported lower prevalence among rural and female participants compared to the present study (Sundarakumar et al., 2023). The high prevalence of neuropsychiatric symptoms in the present study may be due to the sensitivity of the caregiver-based Neuropsychiatric Inventory-Brief in capturing subclinical symptoms (Kaufer et al., 2000). On a global scale, neuropsychiatric symptoms in the elderly are less prevalent but strongly predictive of cognitive decline. A longitudinal study by Kim et al. indicated that symptoms like apathy and anxiety in older adults accelerate cognitive trajectories (Kim et al., 2025). A cross-sectional study by Lyketsos et al. revealed that mild cognitive impairment cohorts reported a high prevalence of at least one symptom, with anxiety, depression and irritability (Lyketsos et al., 2002). These global patterns emphasize the bidirectional nature of neuropsychiatric symptoms and cognition, potentially mediated by neuroinflammation or vascular factors.

The associations between depression, neuropsychiatric symptoms and cognitive performance in the present study corroborate regional and global evidence of interconnected mental health domains in aging. A cross-sectional analysis from India using nationally representative data linked late-life depression to a heightened risk of cognitive decline (Muhammad and Meher, 2021). Another study in Telangana found cognitive impairment in the elderly to be significantly associated with depression (Dhanalakshmi, et al. 2024). A chain mediation model in the elderly revealed that depression directly accounts for 36.85% of cognitive effects, mediated by lifestyle factors (Fan et al., 2024). Globally, studies confirm an association with deficits in processing speed, memory and executive function, with bidirectional effects increasing dementia risk (Liampas et al., 2022). Longitudinal studies show that depressive symptoms in cognitively unimpaired older adults predict amyloid accumulation and faster decline, particularly in episodic memory (Munro et al., 2024). The integration of neuropsychiatric symptoms amplifies these risks, as evidenced by studies linking anxiety and irritability to worse semantic memory and attention (Liampas et al., 2022).

Emerging evidence suggests that the association between depression and cognitive decline may be explained through multiple biological and psychosocial mechanisms. Neuroinflammatory processes, characterized by elevated pro-inflammatory cytokines, have been implicated in both depression and neurodegeneration, potentially accelerating cognitive deterioration (Diniz et al., 2013). Additionally, vascular pathways, including cerebrovascular dysfunction and reduced cerebral perfusion, may contribute to impaired cognitive function in individuals with late-life depression. Psychosocial factors, such as chronic stress, social isolation and reduced cognitive engagement, may further exacerbate cognitive decline by affecting neural plasticity and resilience (Valkanova et al., 2013). The relatively high prevalence observed may partly reflect underlying cultural stigma, social isolation and limited access to mental health services in Gorakhpur. These interconnected mechanisms highlight the complex and multifactorial nature of the depression–cognition relationship in older adults.

### Limitations, future directions and implications

Despite these contributions, the study has certain limitations. The cross-sectional design precludes establishing causal relationships between depression, neuropsychiatric symptoms and cognitive decline; therefore, the observed associations should be interpreted with caution. Additionally, the assessment of neuropsychiatric symptoms relied on caregiver-reported data using the NPI-Brief, which may be subject to measurement bias. Caregiver perceptions can be influenced by factors such as their level of awareness, emotional burden, cultural beliefs and relationship with the participant, potentially leading to underreporting or overreporting of symptoms. Variations in caregiver literacy and understanding of symptoms may further affect reporting accuracy. Similarly, self-reported measures of depression may be affected by recall bias and social desirability, particularly in settings where mental health stigma persists. Although efforts were made to standardize data collection through training and calibration of field investigators, residual bias cannot be entirely ruled out. Additionally, despite using standard generalized linear models to account for non-normal and skewed outcomes, some degree of model misspecification may remain, and, therefore, the possibility of spurious associations cannot be completely excluded. Therefore, the findings should be interpreted in light of these limitations. Future directions should prioritize longitudinal designs to elucidate temporal dynamics and causal pathways, incorporating biomarkers or neuroimaging for mechanistic understanding. Randomized controlled trials targeting depression and neuropsychiatric symptoms may evaluate their impact on cognitive trajectories. Integrating digital tools for remote assessments could enhance accessibility in underserved areas, ultimately fostering healthier aging in India and beyond.

The findings have direct and actionable policy implications for strengthening geriatric mental health services within the primary healthcare system of India, particularly in resource-constrained and rural settings such as Gorakhpur. The integration of routine screening for depression, neuropsychiatric symptoms and cognitive impairment into existing PHC workflows should be institutionalized under the Health and Wellness Centre platform. This can be operationalized by incorporating brief, culturally validated tools such as the ICMR-MUDRA toolbox into standard outpatient geriatric assessments, supported by task-shifting approaches where Accredited Social Health Activists (ASHAs), Auxiliary Nurse Midwives (ANMs), and Community Health Officers are trained to conduct initial screening. Capacity-building policies should prioritize structured training modules for frontline healthcare providers on geriatric mental health, early symptom recognition and referral pathways, ensuring feasibility within existing human resource constraints. Third, community-level interventions, including targeted awareness campaigns and family-based education programs, are essential to address stigma and improve help-seeking behavior, particularly in low-literacy and socioeconomically disadvantaged populations, as reflected in this study. Additionally, policy frameworks should support a stepped-care referral model, linking PHCs with district hospitals and tertiary centers for confirmatory diagnosis and management, thereby ensuring continuity of care. Integration of screening indicators into national programs, such as the National Programme for Health Care of the Elderly (NPHCE), would further enhance scalability and sustainability. Aligning with SDG 3, these policy actions emphasize early detection, integrated care and reduction of untreated mental health burden among older adults, thereby contributing to healthier aging and reduced disability at the population level (Rock et al., 2014; Fan et al., 2024).

## Conclusion

This cross-sectional study, using the culturally adapted ICMR-MUDRA Toolbox, examined the association between geriatric depression, neuropsychiatric symptoms and domain-specific cognitive performance among community-dwelling older adults in Gorakhpur, Uttar Pradesh. The findings demonstrate a high prevalence of both depression and neuropsychiatric symptoms, with agitation and irritability being the most commonly reported manifestations. Importantly, the increasing severity of depression was consistently associated with poorer cognitive performance across multiple domains, with mild depression predominantly affecting executive function and memory, while severe depression showed stronger associations with language and visuospatial impairments. Neuropsychiatric symptoms independently contributed to selective cognitive deficits, particularly in executive and visuospatial functions, even after adjusting for sociodemographic factors.

These findings establish evidence that geriatric depression and neuropsychiatric symptoms are significantly associated with multi-domain cognitive impairment in Indian elderly populations. The high prevalence rates of both depression and neuropsychiatric symptoms, combined with their significant impact on cognitive function. This highlights an immediate need for integrated mental and cognitive health screening and early intervention programs in India. These results provide a foundation for developing targeted interventions to address the interconnected nature of depression and neuropsychiatric symptoms, and cognitive decline in aging populations within the Indian context.

## Supporting information

10.1017/gmh.2026.10246.sm001 Venkatesh et al. supplementary material Venkatesh et al. supplementary material

## Data Availability

All data analyzed during this study are included in the manuscript. Additional data are available from the corresponding author upon reasonable request.

## Supplementary material

The supplementary material for this article can be found at http://doi.org/10.1017/gmh.2026.10246.

## Long descriptions

### Figure 1. Long description

The map is divided into four panels.

Top-left panel: A map of India with state boundaries. Uttar Pradesh is highlighted in red in the North-Central region. A scale bar indicates 0 to 1,100 Kilometers.

Bottom-left panel: A map of Uttar Pradesh showing district boundaries in blue. Gorakhpur District is highlighted in yellow in the Eastern part of the state. A scale bar indicates 0 to 500 Kilometers.

Top-right panel: A map of Gorakhpur District showing internal block boundaries. Several blocks in the Northeast and Central regions are color-coded in shades of green, yellow, orange, and red. A scale bar indicates 0 to 50 Kilometers.

Bottom-right panel: An enlarged view of the study blocks within Gorakhpur. The blocks are labeled as follows:

* Bhathat in light green at the North.

* Chargawan in orange in the Northwest.

* Piprauli in dark orange in the West.

* Khorabar in yellow at the Center.

* Pipraich in red in the Northeast.

* Sardarnagar in medium green in the East.

* Brahmpur in dark green at the South.

A scale bar for this panel indicates 0 to 25 Kilometers. All panels include a North arrow and coordinate grids in degrees, minutes, and seconds.

Navigate back to Figure 1..

### Figure 2. Long description

The flowchart is organized into three vertical stages indicated by blue sidebars.

1. Initial Phase.

- Selection of study blocks and participants using multi-stage random sampling technique. An arrow points right to a box stating Number of blocks selected equals 7, listing Pipraich, Bhathat, Piprauli, Sardarnagar, Brahmpur, Chargawan, and Khorabar.

- Training and calibration of the field investigators for using the I C M R M U D R A toolbox. An arrow points right to a box stating Number of field investigators equals 5.

- Pilot study among 50 participants using the toolbox.

2. M U D R A Toolbox assessment.

- Cognitive assessment using the M U D R A tool box where n equals 1013. An arrow points right to a box detailing Inclusion Criteria: Age greater than 60 years and Permanent residents of the blocks. Exclusion Criteria: Individuals with a history of head injury, infections, stroke, and other neurological, systemic, medical, or psychiatric disorders that can cause cognitive impairment. Individuals with significant hearing or visual impairments that could interfere with the testing were excluded.

3. Assessment of depression and neuropsychiatric symptoms.

- Assessment of geriatric depression and neuropsychiatric symptoms where n equals 1013. An arrow points right to a box listing 1. Geriatric Depression Scale G D S 30 and 2. Neuropsychiatric Inventory N P I caregiver questionnaire.

- The final step at the bottom is Data analysis and Interpretation.

Navigate back to Figure 2..

### Table 1. Long description

The table contains two columns: Demographic profile and n (%).

* Gender: Male 595 (58.7%); Female 418 (41.3%).

* Age: 60 to 64 years 594 (58.6%); 65 to 69 years 296 (29.2%); 70 to 74 years 76 (7.5%); greater than 75 years 47 (4.6%).

* Education: Illiterate 17 (1.7%); Primary school 852 (84.1%); Middle school 37 (3.7%); High school 65 (6.4%); Graduate or above 42 (4.1%).

* Occupation: Clerical/shop/farmer 306 (30.2%); Daily wager 237 (23.4%); House maker 309 (30.5%); Retired 54 (5.3%); Unemployed 107 (10.6%).

* Per capita income (I N R): less than or equal to 3,250 is 338 (33.4); 3,250 to 5,000 is 446 (44.0); greater than 5,000 is 229 (22.6).

* Diet type: Vegetarian 358 (35.3); Nonvegetarian 655 (64.7).

* Tobacco use: Yes 380 (37.5); No 633 (62.5).

* Alcohol consumption: Yes 80 (7.9); No 933 (92.1).

* Hypertension: Yes 112 (11.1); No 901 (88.9).

* Respiratory disease: Yes 52 (5.1); No 961 (94.9).

Navigate back to Table 1..

### Table 2. Long description

The table is organized into four sections: (A) Attention and executive function, (B) Episodic memory, (C) Language test, and (D) Visuo-spatial function. Columns include Mean plus or minus S D, Median with I Q R, Rank sum, H value, and p value.

Section A: Attention and executive function

- Trail making test Part A errors: p equals 0.155. Normal: 3.12 plus or minus 4.56. Severe: 3.87 plus or minus 5.21.

- Trail making test Part B errors: p equals 0.004. Normal: 6.73 plus or minus 6.39. Severe: 6.08 plus or minus 6.00.

- Category fluency test: p equals 0.016. Normal: 12.62 plus or minus 5.71. Severe: 11.52 plus or minus 6.12.

- Phonemic fluency test: p is less than 0.001. Normal: 9.78 plus or minus 3.43. Severe: 8.79 plus or minus 4.34.

Section B: Episodic memory

- Verbal learning test (Free recall): p is less than 0.001. Normal: 17.37 plus or minus 3.46. Severe: 15.00 plus or minus 4.80.

- Verbal learning test (Delayed recall): p is less than 0.001. Normal: 4.73 plus or minus 1.44. Severe: 4.49 plus or minus 1.99.

- Verbal learning test (Delayed recognition): p is less than 0.001. Normal: 13.87 plus or minus 5.64. Severe: 10.86 plus or minus 5.74.

- T N I 93 test Total recall: p is less than 0.001. Normal: 13.28 plus or minus 4.07. Severe: 10.43 plus or minus 4.01.

- T N I 93 test Spatial recall: p is less than 0.001. Normal: 6.33 plus or minus 2.18. Severe: 5.77 plus or minus 2.17.

Section C: Language test

- Picture naming test: p is less than 0.001. Normal: 28.31 plus or minus 3.17. Severe: 26.35 plus or minus 4.96.

- Frenchay aphasia screening test: p is less than 0.001. Normal: 17.58 plus or minus 4.00. Severe: 12.26 plus or minus 4.57.

Section D: Visuo-spatial function

- Modified Taylor complex figure test: p equals 0.029. Normal: 11.10 plus or minus 12.44. Severe: 6.99 plus or minus 8.80.

- Line bisection test: p is less than 0.001. Normal: 15.44 plus or minus 3.68. Severe: 13.59 plus or minus 4.59.

Navigate back to Table 2..

### Table 3. Long description

The table is divided into four sections.

Section A, Attention and executive function.

* Trail Making Test Part A errors: Absent status shows 2.67 plus or minus 3.97; Present status shows 2.52 plus or minus 3.45. p equals 0.846.

* Trail making test Part B errors: Absent status shows 6.26 plus or minus 6.26; Present status shows 5.91 plus or minus 4.85. p equals 0.059.

* Category fluency test: Absent status shows 12.72 plus or minus 5.25; Present status shows 10.73 plus or minus 6.67. p equals 0.001, which is statistically significant.

* Phonemic fluency test: Absent status shows 9.19 plus or minus 4.02; Present status shows 8.85 plus or minus 3.39. p equals 0.389.

Section B, Episodic memory.

* Verbal learning test Free recall: Absent status shows 15.75 plus or minus 4.18; Present status shows 16.57 plus or minus 3.63. p equals 0.006, which is statistically significant.

* Verbal learning test Delayed recall: Absent status shows 4.41 plus or minus 1.66; Present status shows 4.56 plus or minus 1.62. p equals 0.178.

* Verbal learning test Delayed recognition: Absent status shows 11.67 plus or minus 5.67; Present status shows 13.55 plus or minus 6.08. p equals 0.058.

* T N I 93 Test Total recall: Absent status shows 11.47 plus or minus 4.08; Present status shows 12.98 plus or minus 4.32. p equals 0.060.

* T N I 93 Test Spatial recall: Absent status shows 5.70 plus or minus 2.09; Present status shows 6.30 plus or minus 2.42. p equals 0.078.

Section C, Language test.

* Picture naming test: Absent status shows 27.25 plus or minus 4.25; Present status shows 28.00 plus or minus 3.58. p equals 0.066.

* Frenchay aphasia screening test: Absent status shows 14.99 plus or minus 4.76; Present status shows 15.80 plus or minus 4.19. p equals 0.096.

Section D, Visuo-spatial function.

* Modified Taylor complex figure test: Absent status shows 10.43 plus or minus 11.77; Present status shows 7.57 plus or minus 10.72. p equals 0.002, which is statistically significant.

* Line bisection test: Absent status shows 14.44 plus or minus 4.38; Present status shows 14.65 plus or minus 4.00. p equals 0.260.

Navigate back to Table 3..

### Table 4. Long description

The table is organized into four main cognitive domains across the top: (A) Attention and executive function, (B) Episodic memory, (C) Language Test, and (D) Visuo-spatial function.

Sub-tests for these domains include T M T A, T M T B, Category fluency, Phonemic fluency, V L T (Free recall, Delayed recall, and Delayed recognition), T N I minus 93 (total and spatial recall), P N T, F A S T, M T C F, and Line bisection test.

Under Geriatric depression scale category (Model I):

- Mild depression: Significant negative coefficients for T M T B (-0.17), Phonemic fluency (-0.12), all V L T measures (-0.14 to -0.21), T N I minus 93 measures (-0.16), P N T (-0.04), F A S T (-0.17), and Line bisection (-0.08).

- Severe depression: Significant negative coefficients for Category fluency (-0.09), Phonemic fluency (-0.11), V L T Free recall (-0.15) and Delayed recognition (-0.25), T N I minus 93 measures (-0.24 and -0.09), P N T (-0.07), F A S T (-0.36), M T C F (-0.46), and Line bisection (-0.13).

Under Geriatric depression scale category (Model II):

- Mild depression: Similar trends to Model I with slight adjustments, such as T M T B at -0.20 and V L T Delayed recognition at -0.21.

- Severe depression: Significant negative coefficients include T M T B (-0.16), V L T Delayed recognition (-0.22), T N I minus 93 total recall (-0.20), F A S T (-0.30), and M T C F (-0.33).

Under Neuropsychiatric symptoms (Model I):

- Present: Significant negative coefficients for Category fluency (-0.17) and M T C F (-0.32), with a positive significant coefficient for P N T (0.04).

Under Neuropsychiatric symptoms (Model II):

- Present: Significant negative coefficients for Category fluency (-0.13), V L T Delayed recognition (-0.14), F A S T (-0.05), and M T C F (-0.29).

Navigate back to Table 4..
