# Supplementary material for: Relationship between depression, neuropsychiatric symptoms and cognitive profiles of the elderly in India: An assessment using the ICMR-MUDRA toolbox
Source: Glob Ment Health (Camb). 2026 Jun 10;13:e134. doi: 10.1017/gmh.2026.10246 (PMC13373265; doi:10.1017/gmh.2026.10246)
Supplement: Venkatesh et al. supplementary material [file S2054425126102465sup001.docx]

**Supplementary Table 1:** Normality assessment of cognitive performance variables (n = 1,013)

| **Variable** | **W Statistic** | **z-value** | **p-value** |
| --- | --- | --- | --- |
| Trail Making Test Errors – Part A | 0.611 | 13.66 | <0.001 |
| Trail Making Test Errors – Part B | 0.670 | 13.25 | <0.001 |
| Category Fluency Test | 0.944 | 8.86 | <0.001 |
| Phonemic Fluency Test (KaMaPa) | 0.969 | 7.36 | <0.001 |
| Verbal Learning Test (Immediate Recall) | 0.985 | 5.56 | <0.001 |
| Verbal Learning Test (Delayed Recall) | 0.985 | 5.53 | <0.001 |
| Verbal Learning Test (Delayed Recognition) | 0.925 | 9.57 | <0.001 |
| TNI-93 (Total Recall) | 0.915 | 9.90 | <0.001 |
| TNI-93 (Spatial Recall) | 0.991 | 4.21 | <0.001 |
| Picture Naming Test | 0.845 | 11.39 | <0.001 |
| Frenchay Aphasia Screening Test | 0.992 | 4.11 | <0.001 |
| Modified Taylor Complex Figure Test | 0.979 | 6.43 | <0.001 |
| Line Bisection Test | 0.831 | 11.60 | <0.001 |

***Note:*** *Shapiro–Wilk test, p < 0.05*

**Supplementary Figure 1:** Prevalence of neuropsychiatric symptoms and geriatric depression among the participants (n=1013)

**Supplementary Figure 2:** Prevalence of neuropsychiatric symptoms (n=1013)
