# Supplementary material for: Relationship between depression, neuropsychiatric symptoms and cognitive profiles of the elderly in India: An assessment using the ICMR-MUDRA toolbox
Source: Glob Ment Health (Camb). 2026 Jun 10;13:e134. doi: 10.1017/gmh.2026.10246 (PMC13373265; doi:10.1017/gmh.2026.10246)
Supplement: Peer review history

# Reviewer report: Review: Relationship between depression, neuropsychiatric symptoms and cognitive profiles of the elderly in India: An assessment using the ICMR-MUDRA toolbox — R0/PR2

**DOI:** 10.1017/gmh.2026.10246.pr2

Dear Authors,

I congratulate the authors on conducting an important and timely study examining the relationship between geriatric depression, neuropsychiatric symptoms, and cognitive performance among older adults in India using the ICMR-MUDRA toolbox. The manuscript addresses a highly relevant public health issue in the context of rapidly ageing populations and provides valuable community-based evidence. The study design, relatively large sample size, and the use of culturally adapted assessment tools are notable strengths.

However, several aspects of the manuscript require clarification and minor revisions to improve clarity, methodological transparency, and overall readability before it can be considered for publication. My comments below are intended to strengthen the manuscript and enhance its contribution to the literature.

1.Add more recent references (last 3–5 years) on depression and cognitive decline in LMIC settings to strengthen the background literature.

2.Clarify the study setting further by briefly describing whether the selected PHCs were rural, urban, or mixed settings.

3.Add a brief justification for the design effect of three used in the sample size calculation to improve transparency in sampling methodology.

4.Clarify how participants were recruited within PHCs, including whether systematic or consecutive sampling was used at the participant level.

5.Provide additional details on field investigator training, including duration of training and whether standardization procedures were conducted prior to data collection.

6.Clarify whether cognitive test scores were adjusted for education level, as education may significantly influence performance in neuropsychological assessments.

7.Clarify which covariates were included in the adjusted regression model, and provide a brief rationale for selecting these variables.

8.Improve the clarity of the Results section by briefly summarizing key findings from Tables 2–4 rather than presenting only statistical outputs.

9.Add a short paragraph discussing potential mechanisms (e.g., neuroinflammation, vascular pathways, psychosocial stress) linking depression and cognitive decline.

10.Expand the limitations section slightly, particularly addressing the possibility of measurement bias in caregiver-reported neuropsychiatric symptoms.

11.Consider adding brief implications for primary health care practice, particularly how the findings could inform screening strategies at PHCs or community health programs in India.

12.I would suggest bringing Supplementary Figure 1 in the main text as it gives better clarity.

13.The conclusion make it more specific to the study. It should reflect the aim of the study.

Thank you

# Reviewer report: Review: Relationship between depression, neuropsychiatric symptoms and cognitive profiles of the elderly in India: An assessment using the ICMR-MUDRA toolbox — R0/PR3

**DOI:** 10.1017/gmh.2026.10246.pr3

Dear Authors,

The manuscript presents an interesting community-based investigation exploring the relationship between depression, neuropsychiatric symptoms, and cognitive performance among older adults using the ICMR-MUDRA toolbox. However, a few revisions would further improve the clarity and presentation of the manuscript.

Introduction

1.Please briefly define all abbreviations at first use, particularly cognitive tests such as Trail Making Test (TMT), Verbal Learning Test (VLT), Frenchay Aphasia Screening Test (FAST), and Modified Taylor Complex Figure Test (MTCF) to improve readability for a broader audience.

2.Consider adding one or two sentences describing the ICMR-MUDRA toolbox in the Introduction, as the current description mainly appears in the Methods section.

3.The novelty and importance of the ICMR-MUDRA toolbox should be strengthened in the Introduction, particularly how it improves upon existing cognitive assessment tools used in the Indian context.

4.It would also be helpful to clarify whether similar studies examining depression, neuropsychiatric symptoms, and cognitive profiles among elderly populations have been conducted in Gorakhpur or nearby regions. If such studies exist, please reference them to better contextualize the contribution of the present study.

Methods

5.Clarify whether the caregiver responding to the NPI-Brief questionnaire was a family member or another caregiver, as this may influence symptom reporting.

Results

6.The description of the cognitive domains in the Results section could be shortened slightly to improve the overall readability and flow of the section.

7.Ensure that all supplementary figures and tables are clearly referenced in the main text at appropriate locations.

Discussion

8.Consider slightly reducing repetition in the Discussion, particularly where prevalence findings are described multiple times.

Conclusion

9.The conclusion section could briefly highlight the potential policy or programmatic implications, particularly how the findings may inform geriatric mental health screening within primary health care services.

Formatting and References

10.Please ensure consistency in reporting statistical values, particularly spacing and formatting of p-values (e.g., P < 0.001 vs P<0.001).

11.Please check the reference list for duplicate citations, as some references appear more than once (for example, repeated validation studies).

# Reviewer report: Review: Relationship between depression, neuropsychiatric symptoms and cognitive profiles of the elderly in India: An assessment using the ICMR-MUDRA toolbox — R0/PR4

**DOI:** 10.1017/gmh.2026.10246.pr4

Dear Authors,

The manuscript presents an important investigation on the association between geriatric depression, neuropsychiatric symptoms, and cognitive performance among older adults using the ICMR-MUDRA toolbox. The study addresses a relevant public health concern in aging populations. The following suggestions may further improve the overall readability of the manuscript:

• Consider briefly streamlining the abstract, particularly the results section, to improve readability while maintaining the key findings. Some statistical details may be simplified for better flow.

• In the Introduction, a short statement clarifying the research gap addressed by this study in the Indian geriatric population may further strengthen the rationale for conducting the study.

• The objectives at the end of the Introduction could be slightly condensed for clarity and better alignment with the study outcomes.

• In the Methods section, please specify whether all cognitive assessments were administered in a standardized order, as this could influence participant performance during lengthy assessments.

• It may be useful to clarify whether any quality control checks or periodic monitoring of field data collection were performed during the study period.

• In the Data analysis subsection, the description of the composite Trail Making Test score could be simplified to improve readability for readers who may not be familiar with statistical transformations.

• In the Results section, consider adding a brief narrative summary of key patterns observed in Tables 2–4 to guide readers toward the most important findings rather than relying mainly on statistical outputs.

• Some sentences in the Results describing individual cognitive tests may be slightly condensed to improve flow, particularly where similar findings are repeatedly reported across domains.

• In the Discussion, the transition between regional and global literature could be made smoother to improve coherence in the interpretation of findings.

• Consider briefly noting whether cultural or socioeconomic factors in the study region might have influenced the relatively high prevalence of depression observed in the sample.

• The limitations section may benefit from one additional sentence acknowledging potential response bias in self-reported psychological measures among elderly populations.

• The Conclusion could be slightly refined to emphasize the practical implications of integrating mental health and cognitive screening within community-based elderly health programs.

• Please review the manuscript once for minor language consistency and typographical issues, particularly spacing in statistical reporting and uniform presentation of abbreviations.

# Reviewer report: Review: Relationship between depression, neuropsychiatric symptoms and cognitive profiles of the elderly in India: An assessment using the ICMR-MUDRA toolbox — R0/PR5

**DOI:** 10.1017/gmh.2026.10246.pr5

Relationship between depression, neuropsychiatric symptoms and cognitive profiles of the elderly in India: An assessment using the ICMR-MUDRA toolbox

Manuscript ID: GMH-2026-0038

This manuscript entitled “relationship between depression, neuropsychiatric symptoms and cognitive profiles of the elderly in India: An assessment using the ICMR-MUDRA toolbox” presents addresses an important public health issue by examining the association between geriatric depression, neuropsychiatric symptoms, and cognitive performance in an elderly Indian population. Using a cross-sectional design and a large community-based sample (n=1,013), the study reports a high prevalence of depression (63.3%) and neuropsychiatric symptoms (78.5%). The findings demonstrate a significant decline in cognitive performance across multiple domains with increasing depression severity, with differential domain-specific associations observed for mild and severe depression. Neuropsychiatric symptoms were also found to independently affect cognitive outcomes. Overall, the study highlights the need for integrating mental health screening and early interventions into routine geriatric care.

Comments to the Author

1. Clearly define the concepts of depression, cognitive profile, and neuropsychiatric symptoms in the manuscript, supported by authentic and credible references.

2. Ensure that the research is aligned with at least one Sustainable Development Goal (SDG). This alignment should be clearly incorporated and justified in both the Introduction and Discussion sections.

3. A comprehensive literature review should be included, highlighting key findings from previous studies. It should demonstrate how the present study is meaningful and impactful at the global, national, and local levels. Additionally, a clear justification must be provided for selecting Gorakhpur, Uttar Pradesh (India), as the study area.

4. On Page 3, Line 38 (“The present study … in Gorakhpur, Uttar Pradesh”), the use of “knowledge gap” as a rationale is too generic and insufficient. The research gap should be more robust and well-justified, derived from a structured literature review. It should also explain why Gorakhpur is a significant study area compared to other regions in India.

5. Detailed psychometric properties of the measures used for depression, cognitive profile, and neuropsychiatric symptoms should be provided, along with proper references.

6. The study employs non-parametric tests instead of parametric tests. A clear justification should be given explaining why non-parametric methods are more appropriate for this study.

7. The use of a Generalized Linear Regression Model (GLRM) to determine associations is relatively basic and may lead to spurious conclusions. The methodological choice should be justified, and its limitations should be acknowledged.

8. On Page 3, Line 9 (“Additionally, linguistic diversity … differences (Lakshiminarayanan et al., 2022)”), the rationale for selecting the toolbox in the Indian context is well-stated. However, the study examines associations between cognitive profile, depression, and neuropsychiatric symptoms using GLRM without incorporating latent variables such as socio-economic, demographic, and health-behavioral factors. Given India’s diverse population, it would strengthen the analysis to include these latent variables and apply more advanced models such as logistic or multinomial logistic regression. Furthermore, the study could explore how these associations vary by gender and place of residence.

9. The study should examine regional patterns of cognitive profile, depression, and neuropsychiatric symptoms, and attempt to identify region-specific triggering factors behind these phenomena.

10. The study should propose relevant, practical, and impactful policy recommendations. Without strong policy implications, the study’s usefulness is limited. The suggested policies should reflect ground realities and be adaptable to the specific context of the study area.

# Editor report: Recommendation: Relationship between depression, neuropsychiatric symptoms and cognitive profiles of the elderly in India: An assessment using the ICMR-MUDRA toolbox — R0/PR6

**DOI:** 10.1017/gmh.2026.10246.pr6

**Affiliations:** Heilbrunn Department of Population and Family Health, Columbia University, United States

Dear Dr. Venkatesh, 

All reviewers agree that this study addresses a highly relevant public health issue in the context of rapidly aging populations. Reviewers request major revisions to improve methodological transparency, clarity, and presentation of findings. Please see reviewers' comments for further details. I recommend acceptance pending these major revisions. 

# Editor report: Decision: Relationship between depression, neuropsychiatric symptoms and cognitive profiles of the elderly in India: An assessment using the ICMR-MUDRA toolbox — R0/PR7

**DOI:** 10.1017/gmh.2026.10246.pr7

**Affiliations:** London School of Hygiene & Tropical Medicine, United Kingdom of Great Britain and Northern Ireland

No accompanying comment.

# Reviewer report: Review: Relationship between depression, neuropsychiatric symptoms and cognitive profiles of the elderly in India: An assessment using the ICMR-MUDRA toolbox — R1/PR9

**DOI:** 10.1017/gmh.2026.10246.pr9

Revised version seems to be satisfactory for publication.

# Reviewer report: Review: Relationship between depression, neuropsychiatric symptoms and cognitive profiles of the elderly in India: An assessment using the ICMR-MUDRA toolbox — R1/PR10

**DOI:** 10.1017/gmh.2026.10246.pr10

I am satisfied with the corrections. I recommend Publication.

# Reviewer report: Review: Relationship between depression, neuropsychiatric symptoms and cognitive profiles of the elderly in India: An assessment using the ICMR-MUDRA toolbox — R1/PR11

**DOI:** 10.1017/gmh.2026.10246.pr11

The manuscript addresses an important and relevant topic concerning the relationship between depression, neuropsychiatric symptoms, and cognitive profiles among the elderly population in India using the ICMR-MUDRA toolbox. The study is well-structured, methodologically sound, and provides valuable insights for geriatric mental health research. The analyses are appropriate, and the findings are clearly presented and discussed in the context of existing literature.

I appreciate the authors’ efforts in revising the manuscript and addressing previous concerns. The manuscript is suitable for publication after minor editorial revisions related to language clarity and formatting.

# Reviewer report: Review: Relationship between depression, neuropsychiatric symptoms and cognitive profiles of the elderly in India: An assessment using the ICMR-MUDRA toolbox — R1/PR12

**DOI:** 10.1017/gmh.2026.10246.pr12

Dear Authors,

I have reviewed the revised version of the manuscript entitled “Relationship between depression, neuropsychiatric symptoms and cognitive profiles of the elderly in India: An assessment using the ICMR-MUDRA toolbox.”

The authors have adequately addressed the comments and suggestions provided during the review process. The revisions made have improved the clarity and overall quality of the manuscript. I appreciate the authors’ efforts in responding carefully to the reviewer comments and incorporating the necessary changes in the revised version.

In my opinion, the manuscript is now suitable for publication.

# Editor report: Recommendation: Relationship between depression, neuropsychiatric symptoms and cognitive profiles of the elderly in India: An assessment using the ICMR-MUDRA toolbox — R1/PR13

**DOI:** 10.1017/gmh.2026.10246.pr13

**Affiliations:** Heilbrunn Department of Population and Family Health, Columbia University, United States

Dear Authors, 

The reviewers have noted that their comments and suggestions have been adequately addressed during the review process. The manuscript is now suitable for publication. 

Thank you.

# Editor report: Decision: Relationship between depression, neuropsychiatric symptoms and cognitive profiles of the elderly in India: An assessment using the ICMR-MUDRA toolbox — R1/PR14

**DOI:** 10.1017/gmh.2026.10246.pr14

**Affiliations:** London School of Hygiene & Tropical Medicine, United Kingdom of Great Britain and Northern Ireland

No accompanying comment.
